# Supplementary material for: The mouse KLF1 Nan variant impairs nuclear condensation and erythroid maturation
Source: PLoS One. 2019 Mar 28;14(3):e0208659. doi: 10.1371/journal.pone.0208659 (PMC6438607; doi:10.1371/journal.pone.0208659)
Supplement: S1 File — (DOCX) [file pone.0208659.s001.docx]

**The mouse KLF1 *Nan* variant impairs erythroid maturation during fetal development**

Ileana Cantú, Harmen J.G. van de Werken, Nynke Gillemans, Ralph Stadhouders, Steven Heshusius, Alex Maas, Fatemehsadat Esteghamat, Zeliha Ozgur, Wilfred F.J. van IJcken, Frank Grosveld, Marieke von Lindern, Sjaak Philipsen and Thamar B. van Dijk

**Supplementary materials and methods**

**FigA-D**

**Supplementary materials and methods**

**Mice**

All animal studies were approved by the Erasmus MC Animal Ethics Committee. The mouse strains used were *Klf1* *Nan* mutant ([1](#_ENREF_1)) and *Klf1* knockout ([2](#_ENREF_2)). Genotyping was performed by polymerase chain reaction (PCR) using DNA isolated from toe biopsies. Primers are listed below. For *Klf1* *Nan* genotyping, the PCR product was digested with DpnII. Embryos were collected at E12.5, E13.5, E14.5 and E18.5; tail DNA was used for genotyping.

*Klf1 Nan*

Fw: 5’CTGCAGGATTGCAGCTGTAGATAC3’

Rv: 5’ AGTCCTTGTGCAGGATCACTCAGA3’

Approximately 340 bp PCR product for the wild type allele, and 240 + 100 bp for the *Nan* allele after DpnII digestion.

*Klf1* knock-out

Fw1: 5’TTGCCGTTTTGCTTTGCCTG3’

Fw2: 5’CGTTGGCTACCCGTGATATTG3’

Rv: 5’GAAGTCCTCCTGGGTGTCCA3’

Approximately 250 bp PCR product for the wild type allele and 270 bp for the knockout allele

**RNA isolation and RT-qPCR analyses**

RNA was extracted using TRI reagent (Sigma-Aldrich). To synthesize cDNA, 2 μg of RNA were used together with oligo dT (Invitrogen), RNase OUT (Invitrogen), and SuperScript reverse transciptase II (Invitrogen) in a total volume of 20 μL for 1 hour at 42 degrees. 0.2 μL of cDNA was used for amplification by RT-qPCR. Amplification was performed on a CFX96 Touch Real-Time PCR Detection System (Biorad) with the primers listed below using Platinum Taq DNA polymerase (Invitrogen) and 40 cycles consisting of 95°C for 30 sec, 60°C for 1 min. Specific polymerase chain reaction (PCR) product accumulation was monitored by SYBR Green dye fluorescence (Sigma-Aldrich). Ct values obtained for actin expression were used for normalization. Dissociation curves assessed the homogeneity of PCR products.

Xpo7 exon 1b

Fw: 5’GGGCTGTTGGTGAATTCAACC3’

Rv: 5’TTGAGACATGCGGGACTCAG3’

Actin-b

Fw: 5’GATTACTGCTCTGGCTCCT3’

Rv: 5’TGGAAGGTGGACAGTGAG3’

Klf1

Fw: 5’CAGCTGAGACTGTCTTACCC3’

Rv: 5’AATCCTGCGTCTCCTCAGAC3’

**Flow cytometry analysis**

Single-cell suspensions collected from fetal livers or I/11 cell cultures were washed twice with PBS and then resuspended in PBS containing 1% (w/v) bovine serum albumin and 1 mM EDTA. Approximately 10^6^ cells were incubated for 30 minutes at room temperature with Kit-PE (553355, BD Biosciences, dilution 1:400), CD44-APC (559250, BD Biosciences, dilution 1:1600), CD71-BV421 (562716; BD Biosciences, dilution 1:400) and Ter119-APC (17-5921-82; eBiosciences, dilution 1:400) antibodies and in a final volume of 100 µl. The cells were washed, and living cells were distinguished negatively by 7-aminoactinomycin D staining (A1310; Invitrogen). Cells were measured on a Fortessa instrument (BD Biosciences), and data were analyzed with FlowJo software v10 (Tree Star). E18.5 fetal liver cells were sorted by using CD71-FITC (553266; BD Biosciences) antibody and MACS separation columns according to the manufacturer’s instructions (Miltenyi Biotec).

For enucleation analysis, cells were stained with 10 µg/ml Hoechst 33342 (H3570, Invitrogen), in addition to Ter119-APC (17-5921-82; eBiosciences, dilution 1:100) antibody, for 15 min at room temperature.

The nuclear area of transduced I/11 cells was measured and analyzed on an ImageStreamX Mark II Imaging Flow Cytometer (Amnis). The cells were stained with CD71-FITC (333151, BD Biosciences, dilution 1:200) and 5 μM DRAQ5 (Thermo Fischer).

**Cell morphology**

Cell morphology was analyzed using cytospins stained with May Grünwald-Giemsa (Medion Diagnostic) and O-dianisidine (Sigma-Aldrich) ([3](#_ENREF_3)). Pictures were taken with an Olympus BX40 microscope (40x objective, NA 0.65) equipped with an Olympus DP50 CCD camera and Viewfinder Lite 1.0 acquisition software.

Nuclear size was measured with DAPI staining (P-36931, Life technologies). Pictures were taken with Leica DMRBE microscope (40x objective, NA 0.65) equipped with Hamamatsu ORCA-ER digital camera and Hokawo Imaging Software v 2.6. Images were analyzed using Fiji ([4](#_ENREF_4)).

**RNA-sequencing and analysis**

RNA-seq was performed according to manufacturer’s instructions (Illumina), as described([5](#_ENREF_5)). The sequenced reads were mapped against the mouse genome build mm10 using TopHat 2.0.6 ([6](#_ENREF_6)) with the transcriptome gene annotation of Ensembl v73 ([7](#_ENREF_7)). Raw counts were generated with HTSeq-count version 0.6.0. using the settings -m union -s no -a 20 ([8](#_ENREF_8)) with the Ensembl 73 gene annotation. The counted data were normalized by the size factor of the libraries using DESeq2 R package ([9](#_ENREF_9)) and, subsequently, converted to Transcripts Per Million (TPM) as described in ([10](#_ENREF_10)). The differentially expressed genes were called using a generalized linear negative binomial model that controlled for the effect of each litter. The calculations were performed by the DESeq2 R package. After using the Wald significance test the False Discover Rates (FDR)/adjusted p-values were calculated with the Benjamini Hochberg method ([11](#_ENREF_11)). Threshold value for differentially expressed genes was set to a FDR <0.01 with an absolute fold change of 1.5. After blind variance stabilizing transformation of the normalized counts ([9](#_ENREF_9)), the differentially expressed genes were used to calculate the pairwise Spearman's rank correlation coefficient (r*_s_*) matrices and the subsequent dissimilarity matrices (1 – r_s_) for both genes and RNA-Seq samples. The Euclidean distances of the dissimilarity matrices were used to apply hierarchical clustering with complete linkage. The normalized gene expression data were scaled for each row (Z-score) and, subsequently, plotted in a heat map using the R statistical package version 3.2.0 (R-Core-Team: R: A language and environment for statistical computing. Vienna, Austria: R Foundation for Statistical Computing; 2015).

**Chromosome Conformation Capture Combined with high-throughput Sequencing (4C-seq) and data analysis**

4C-seq experiments were carried out as described ([12](#_ENREF_12), [13](#_ENREF_13)). Briefly, 4C-seq template was prepared from E13.5 fetal liver or fetal brain cells. In total, between 1 and 8 million cells were used for analysis. The cells were cross-linked with 2% formaldehyde in PBS/10% FBS for 10 min at room temperature. After quenching the reaction with 0.125M glycine, cells were lysed in lysis buffer (50mM Tris pH 7.5, 150mM NaCl, 5mM EDTA, 0.5% NP-40, 1% Triton X-100, and protease inhibitor mixture Complete (Roche)). The cross-linked DNA was digested with DpnII as the primary restriction enzyme and BfaI as a secondary restriction enzyme. Viewpoint-specific primers from the 4C-seq primer database ([14](#_ENREF_14)) with additional Illumina adapter sequences as overhangs were used to generate sequencing libraries. Primer sequences are listed below:

Canonical promoter

Fw: 5’TCACTGAGTCAATTTTGCTG3’

Rv: 5’ TAGGCCAGACTACTCAGATC 3’

Erythroid specific exon 1b

Fw: 5’GGTCATGTTTCCTTAGCTCTG3’

Rv: 5’GCTGAGGAGATGATGGATC3’

Eight PCR reactions were carried out using 100 ng of 4C template per reaction. The products were pooled and purified using the QIAquick PCR purification kit (Qiagen). Samples were subjected to deep sequencing using an Illumina HiSeq2000 platform. Two replicate experiments were sequenced for each genotype and viewpoint.

4C-seq data analysis was performed as described in ([13](#_ENREF_13), [14](#_ENREF_14)). In brief, the 4C-seq reading primer sequences with their barcodes were used to de-multiplex the reads and to trim from the 5’-end to the first restriction enzyme recognition site. The sequences were mapped, while ignoring quality scores of the read bases and not allowing for a mismatch, to a database of digested genome fragment-ends using the mouse reference genome build mm10. All the 4C-seq samples passed the quality control threshold values as described in ([13](#_ENREF_13)). We normalized the data taking the library size and 4C-seq fragment-end types ([13](#_ENREF_13), [14](#_ENREF_14)) into account. The 4C-seq contact profiles were generated after the median value of the biological replicates (wildtype fetal liver, fetal brain and *Nan* fetal liver, respectively) was calculated. To smoothen the data we applied a running trimmed (10%) mean approach using 21 fragment-ends in a single window. We further determined differentially contact frequencies between 1) wild type fetal liver and brain and between 2) *Nan* fetal liver and wildtype fetal liver (data not shown). Since 4C-seq data don't follow a specific distribution are biased for each fragment-end differently, we used a non-parametric approach to test for statistical significance between two phenotypes. We therefore ranked all normalized data for each fragment-end independently, and set ties to the minimal value. Subsequently, we binned, along the locus, the ranks of 21 fragment-ends and calculated the *m* x *n* rank frequency matrix (*m* = 2; *i.e.* the number of different phenotypes; *n* = number of samples). We merged the frequency matrix columns into a 2 x 2 matrix, based on the order and the number of samples of each phenotype e.g. if a phenotype that is ranked first, based on the enumeration of the rank frequencies multiplied by the rank number, was sampled three times, the first three columns of the *m* x *n* matrix are merged by adding up the row values, and ,subsequently, the other columns, representing the second phenotype, are merged similarly resulting in a 2 x 2 matrix. A *Χ^2-^*-test was applied to this 2 x 2 contact frequency matrix. The *p-values* were corrected for multiple hypotheses testing using the Benjamini Hochberg method ([11](#_ENREF_11)) and areas with significant contact differences are indicated in gray in Fig 4B,C. The R statistical package version 3.2.0 was used for the statistical calculations and for generating the 4C-seq contact plots (R-Core-Team: R: A language and environment for statistical computing. Vienna, Austria: R Foundation for Statistical Computing; 2015). Gviz was used for plotting the annotation data (Florian Hahne, Steffen Durinck, Robert Ivanek, Arne Mueller, Steve Lianoglou, Ge Tan and Lance Parsons. Gviz: Plotting data and annotation information along genomic coordinates. R package version 1.12.1).

**References to supplementary materials and methods**

1. Lyon MFG, P.H.; Loutit, J.F., Peters, J. . Dominant hemolytic anemia. Mouse News Letter. 1983;68:68.

2. Nuez B, Michalovich D, Bygrave A, Ploemacher R, Grosveld F. Defective haematopoiesis in fetal liver resulting from inactivation of the EKLF gene. Nature. 1995;375(6529):316-8.

3. Beug H, Palmieri S, Freudenstein C, Zentgraf H, Graf T. Hormone-dependent terminal differentiation in vitro of chicken erythroleukemia cells transformed by ts mutants of avian erythroblastosis virus. Cell. 1982;28(4):907-19.

4. Schindelin J, Arganda-Carreras I, Frise E, Kaynig V, Longair M, Pietzsch T, et al. Fiji: an open-source platform for biological-image analysis. Nat Methods. 2012;9(7):676-82.

5. Meinders M, Kulu DI, van de Werken HJ, Hoogenboezem M, Janssen H, Brouwer RW, et al. Sp1/Sp3 transcription factors regulate hallmarks of megakaryocyte maturation and platelet formation and function. Blood. 2015;125(12):1957-67.

6. Kim D, Pertea G, Trapnell C, Pimentel H, Kelley R, Salzberg SL. TopHat2: accurate alignment of transcriptomes in the presence of insertions, deletions and gene fusions. Genome Biol. 2013;14(4):R36.

7. Flicek P, Amode MR, Barrell D, Beal K, Billis K, Brent S, et al. Ensembl 2014. Nucleic Acids Res. 2014;42(Database issue):D749-55.

8. Anders S, Pyl PT, Huber W. HTSeq--a Python framework to work with high-throughput sequencing data. Bioinformatics. 2015;31(2):166-9.

9. Love MI, Huber W, Anders S. Moderated estimation of fold change and dispersion for RNA-seq data with DESeq2. Genome Biol. 2014;15(12):550.

10. Wagner GP, Kin K, Lynch VJ. Measurement of mRNA abundance using RNA-seq data: RPKM measure is inconsistent among samples. Theory Biosci. 2012;131(4):281-5.

11. Benjamini Y, Hochberg Y. Controlling the False Discovery Rate - a Practical and Powerful Approach to Multiple Testing. J R Stat Soc B. 1995;57(1):289-300.

12. Stadhouders R, Kolovos P, Brouwer R, Zuin J, van den Heuvel A, Kockx C, et al. Multiplexed chromosome conformation capture sequencing for rapid genome-scale high-resolution detection of long-range chromatin interactions. Nat Protoc. 2013;8(3):509-24.

13. van de Werken HJ, de Vree PJ, Splinter E, Holwerda SJ, Klous P, de Wit E, et al. 4C technology: protocols and data analysis. Methods Enzymol. 2012;513:89-112.

14. van de Werken HJ, Landan G, Holwerda SJ, Hoichman M, Klous P, Chachik R, et al. Robust 4C-seq data analysis to screen for regulatory DNA interactions. Nat Methods. 2012;9(10):969-72.

15. Su MY, Steiner LA, Bogardus H, Mishra T, Schulz VP, Hardison RC, et al. Identification of biologically relevant enhancers in human erythroid cells. J Biol Chem. 2013;288(12):8433-44.

**Supplementary Figures**

**FigA**


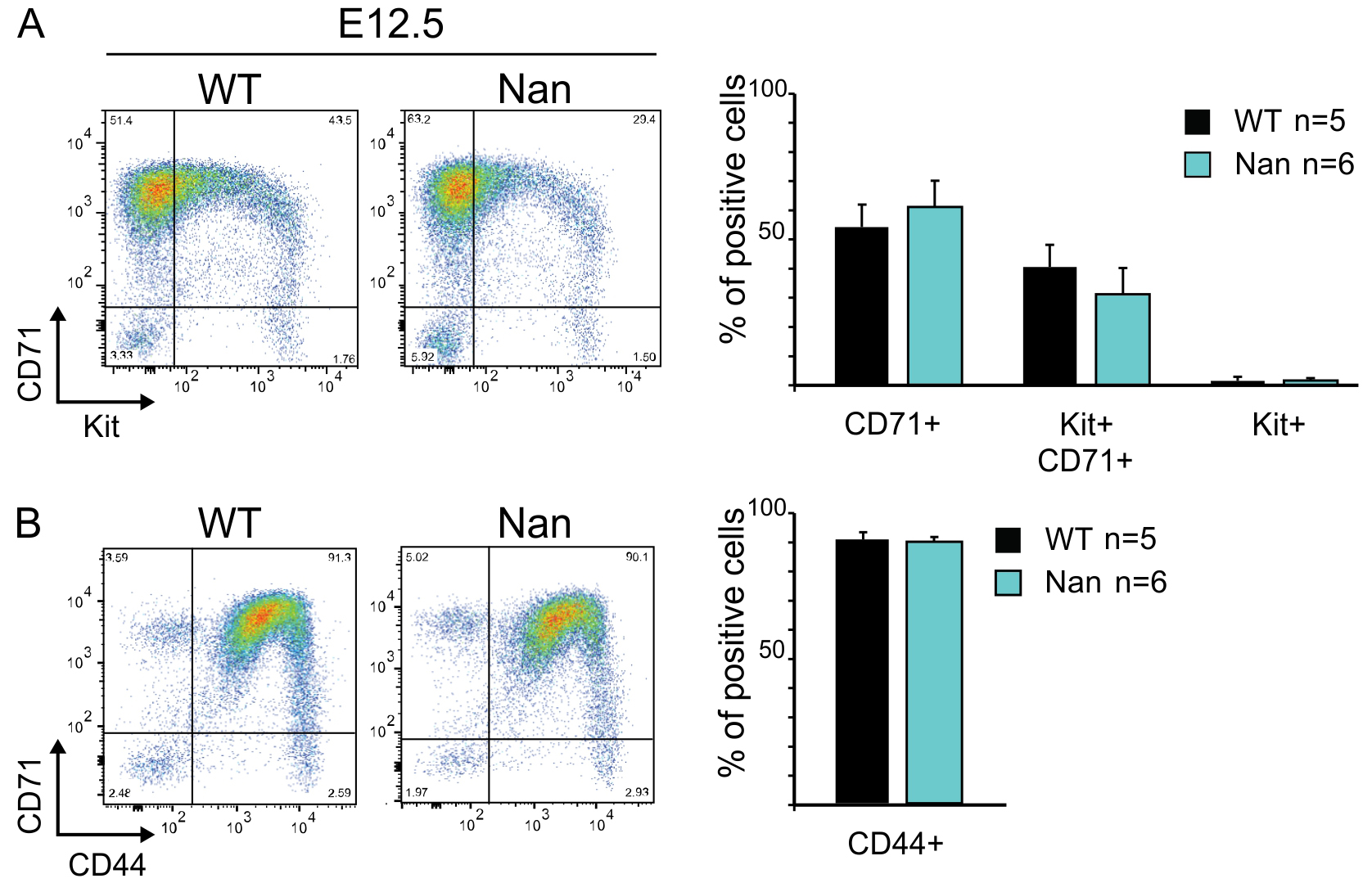


**FigA.** **Flow cytometry analysis of fetal liver cells.** (A) Example of flow cytometry profiles of CD71 and Kit staining of wildtype and *Nan* E12.5 fetal livers and quantification of CD71+, CD71+ Kit+ and Kit+ populations. n indicates the number of embryos. (B) Example of flow cytometry profiles of CD71 and CD44 staining of wildtype and *Nan* E12.5 fetal livers and quantification of C44+population. n indicates the number of embryos.

**FigB**


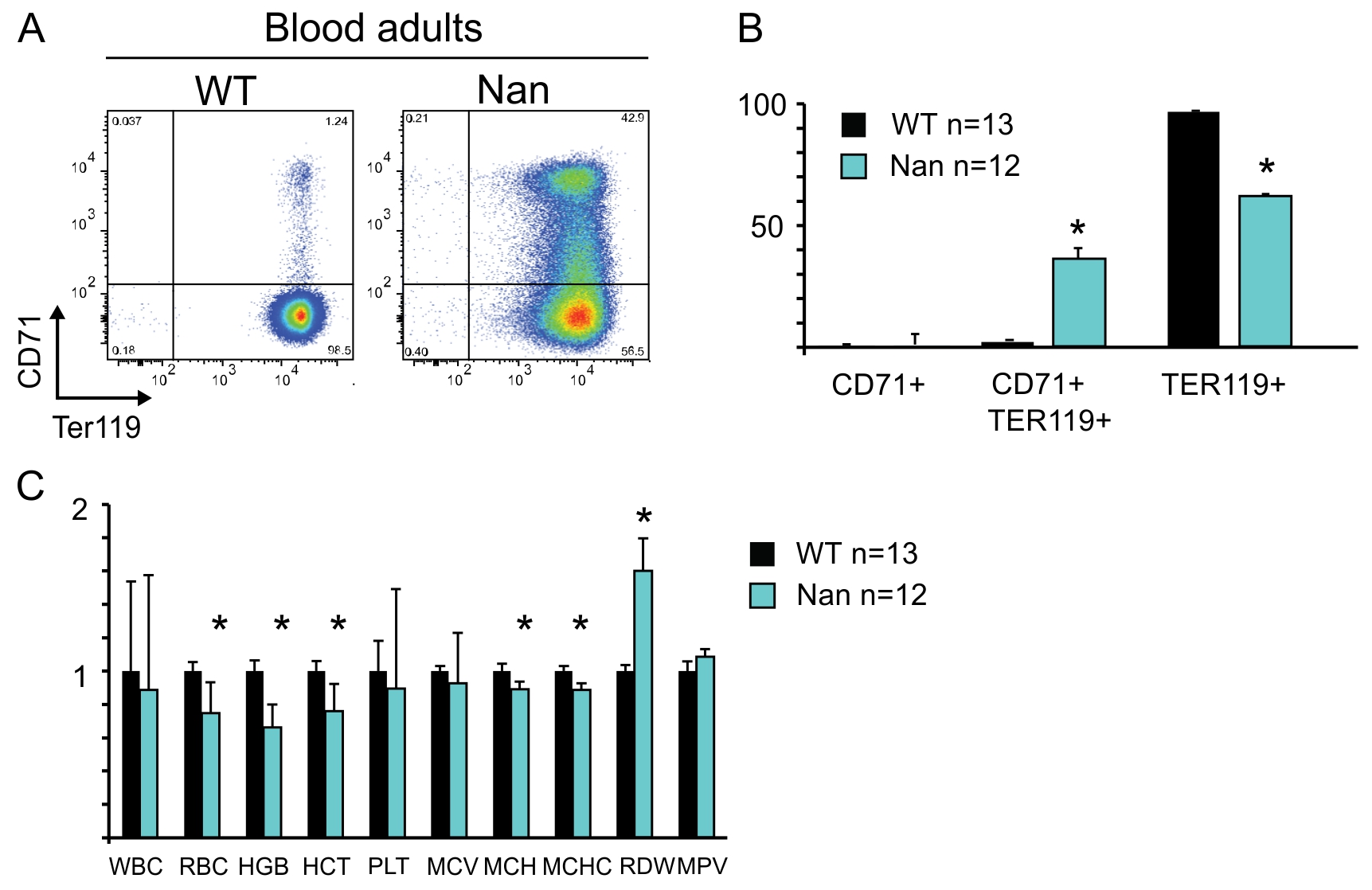


**FigB.** **Flow cytometry analysis and complete blood count of peripheral blood.** (A) Example of flow cytometry profiles of CD71 and Ter119 staining of wildtype and *Nan* adult blood. (B) Quantification of CD71+, CD71+ Ter119+ and Ter119+ populations. n indicates the number of mice. * indicates *p value* <0.01. (C) Hematologic parameters of wildtype and *Nan* adult mice. * indicates *p value* <0.01. WBC, with blood cells; RBC, red blood cells; HGB, hemoglobin; HCT, hematocrit; PLT, platelets; MCV, mean corpuscular volume, MCH, mean corpuscular hemoglobin, MCHC, mean corpuscular hemoglobin concentration, RDW, red cell distribution width; MPV, mean platelet volume.

**FigC**


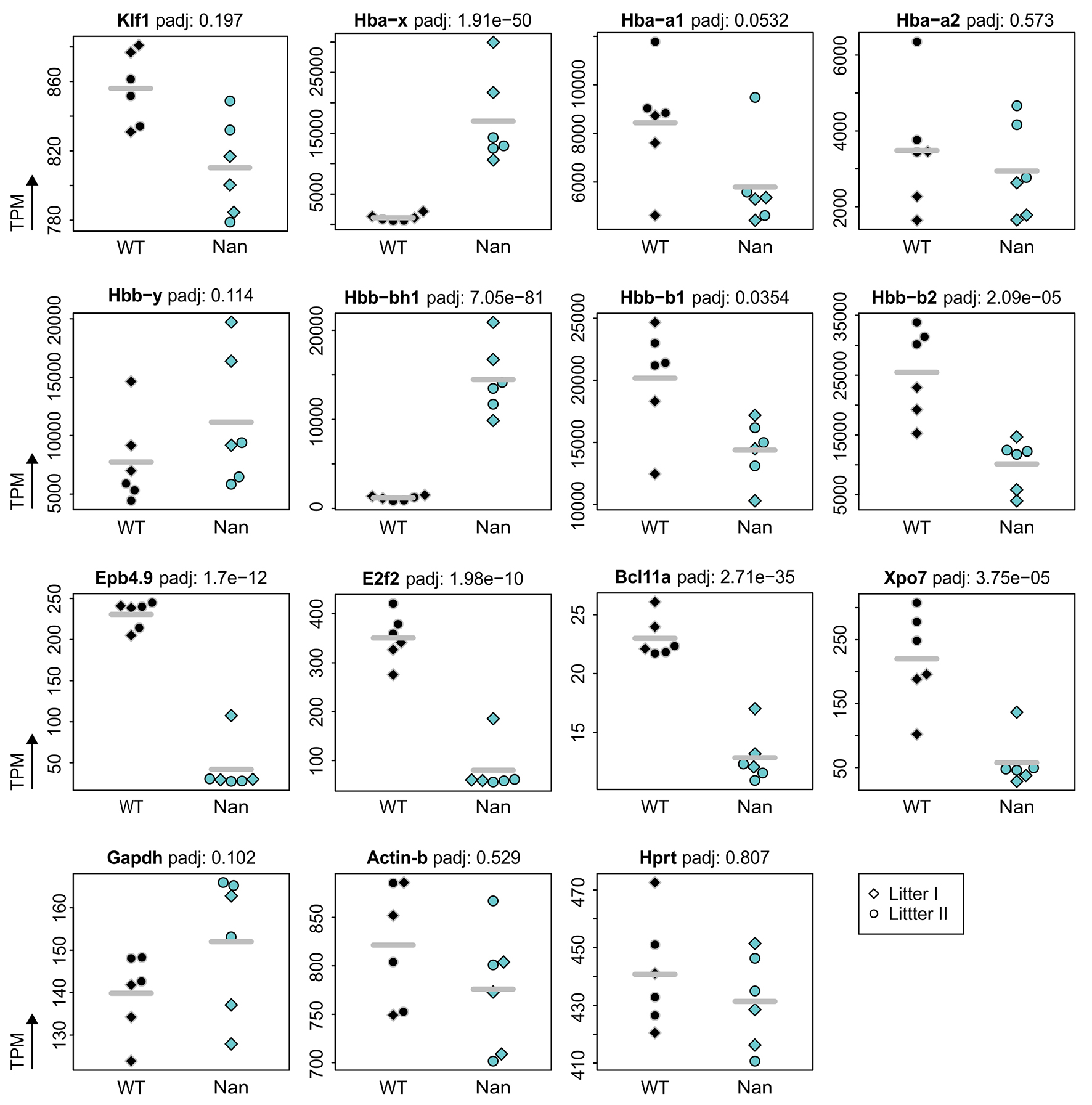


**FigC.** **Gene expression analysis by RNA-seq**. Gene expression levels were determined by RNA-seq using RNA isolated from wildtype (black) and *Nan* (cyan) E12.5 mouse fetal liver cells. Levels of selected erythroid-specific genes combined with the controls Gapdh, Actin-b and Hprt are shown in Transcripts Per Million (TPM) with adjusted *p-values* (*padj*).

**FigD**


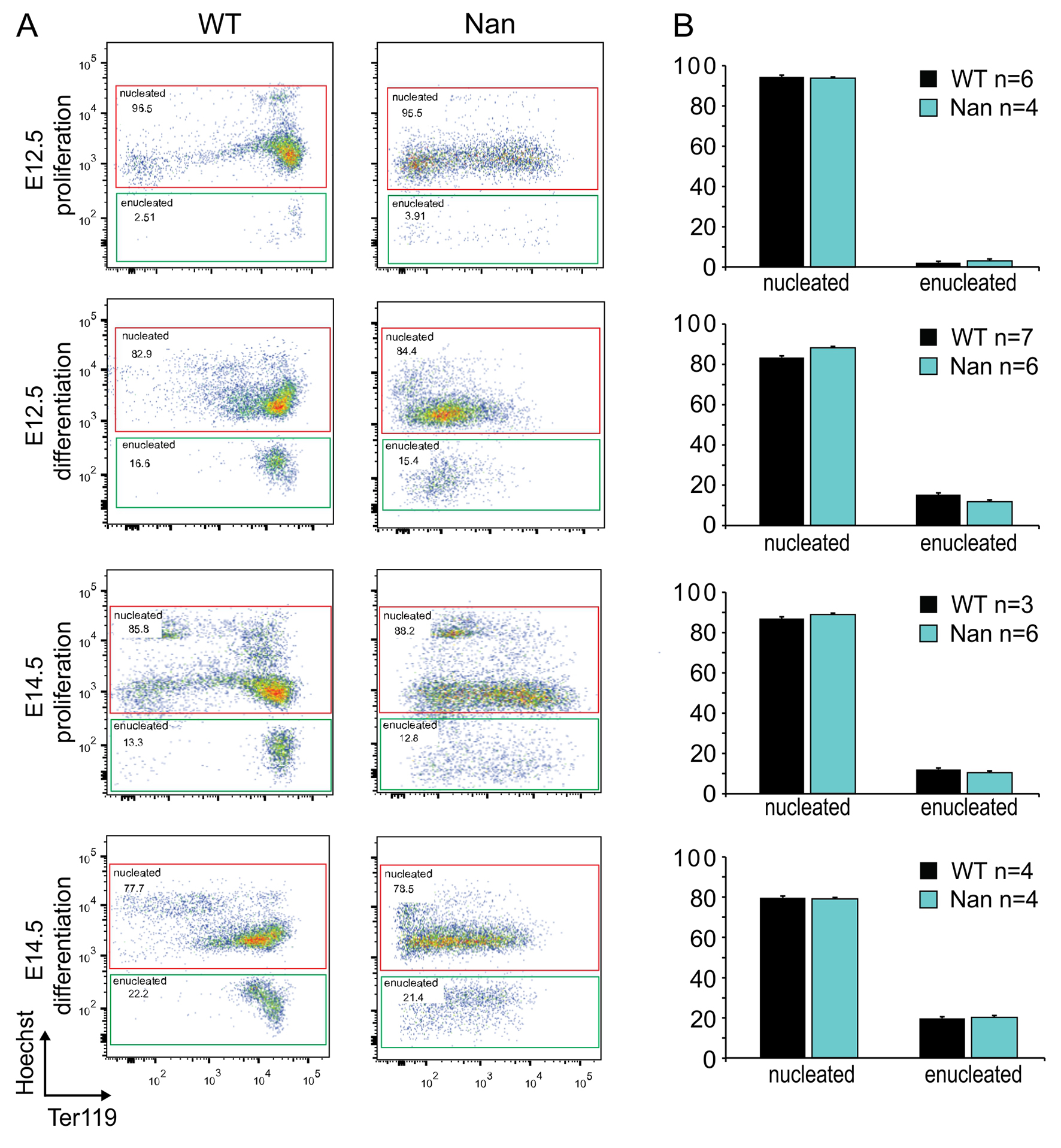


**FigD.** **Analysis of nucleated cells**. (A) Gating strategies of Hoechst- and Ter119-stained E12.5 and E14.5 fetal liver cells in proliferative and differentiation medium. Red, nucleated population (Hoechst+); Green, enucleated population (Hoechst-). (B) Quantification of the number of nucleated (Hoechst+) and enucleated (Hoechst-) cells. n indicates the number of embryos.
